# Occurrence of Waterborne Protozoa in a Full-Scale Drinking Water Treatment Plant in China by High-Throughput Screening and Genotyping

**DOI:** 10.1021/envhealth.5c00863

**Published:** 2026-05-06

**Authors:** Huican Zhang, Ziming Han, Fuming Duan, Shumin Xiao, Min Yang, Yu Zhang

**Affiliations:** † Henan Institute of Advanced Technology, Zhengzhou University, Zhengzhou 450003, China; ‡ Key Laboratory of Environmental Aquatic Chemistry, State Key Laboratory of Regional Environment and Sustainability, Research Center for Eco-Environmental Sciences, 12381 Chinese Academy of Sciences, Beijing 100085, China; § School of Environmental and Municipal Engineering, 74774 Tianjin Chengjian University, Tianjin 300384, China; ∥ National Engineering Research Center of Industrial Wastewater Detoxication and Resource Recovery, Research Center for Eco-Environmental Sciences, Chinese Academy of Sciences, Beijing 100085, China; ⊥ University of Chinese Academy of Sciences, 100049 Beijing, China

**Keywords:** *Cryptosporidium parvum*, *Enterocytozoon bieneusi*, HT-qPCR, backwash water

## Abstract

This study investigates potential risk points for waterborne protozoa in a full-scale drinking water treatment plant using high-throughput screening and genotyping. Among 19 protozoan targets of high-throughput quantitative PCR (HT-qPCR), 11 were positive, with *Cryptosporidium* (22.2%) and *Enterocytozoon bieneusi* (22.2%) exhibiting the highest detection rates. Three and two protozoan species were detectable in the source water of the reservoir and plant influents, respectively; while no protozoa were detected above the detection limit in plant effluents, as confirmed by both HT-qPCR and an immunofluorescence assay targeting*Cryptosporidium* oocysts and *Giardia* cysts. Notably, backwash water (from the sand filter and activated carbon filter) and sediment (from the sedimentation tank) samples exhibited higher prevalence and abundance of protozoa. Particularly, backwash water from the sand filter contained eight protozoan targets, with a total abundance of 1.5 × 10^5^ copies/g. Further nested PCR-based genotyping revealed that detected *Cryptosporidium parvum* belonged to *gp60* subtype IIdA19G1 and *E. bieneusi* was identified as genotype AAE1, suggesting that *C. parvum* may pose a threat to human health. Overall, the current drinking water treatment process ensures effective protozoan removal in the effluent, but the backwash water and sediment pose potential risk. This study suggested enhancing monitoring and implementing additional measures for key risk points to optimize the prevention and control of waterborne protozoa.

## Graphical abstract

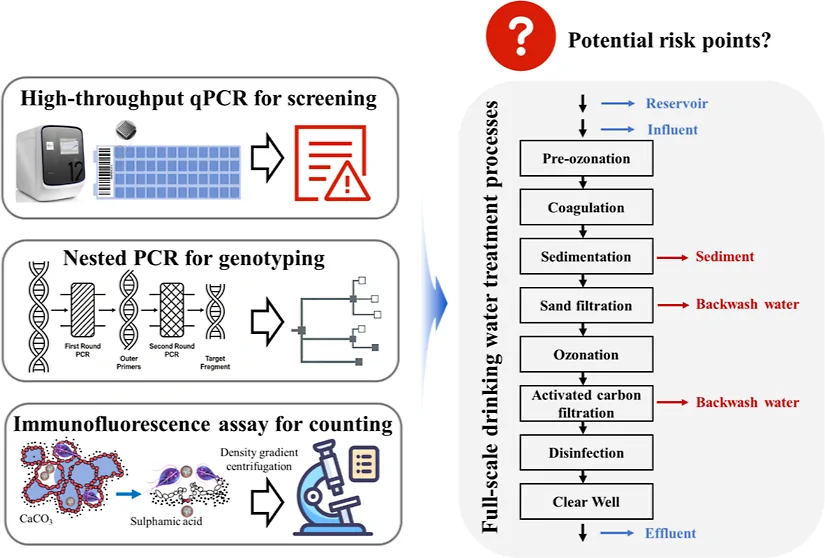

## Introduction

Waterborne protozoa remain a persistent challenge for drinking water safety, because their transport and removal efficiency within treatment systems determine the residual risk in treated water.^1,2^ Global reports indicated that between 2017 and 2022, parasitic protozoa caused 416 waterborne outbreaks worldwide. *Cryptosporidium* accounted for 77.4% of outbreaks (322 cases), while *Giardia* was identified as the etiological agent in 17.1% (71 cases); among them, 71 (17.06%) outbreaks were found to be connected to drinking water.^3^ *Cryptosporidium* was a leading cause of diarrhea-related morbidity and mortality worldwide,^4–6^ with malnutrition posing a particularly critical concern in developing countries. Cryptosporidiosis was prevalent among children and was often associated with persistent diarrhea, malnutrition, growth stunting, and cognitive impairment.^7^ *Giardia* is a flagellate protozoan parasite infecting the upper intestinal tract of humans and a wide range of other mammals worldwide. The US EPA requires testing for *Cryptosporidium* and *Giardia* and mandated the removal of 99.9% of these pathogens from drinking water.^8^ China’s drinking water quality standards require that the concentration of (oo)­cysts of *Cryptosporidium* and *Giardia* in the effluent should be less than 1 per 10 L (GB 5749-2022).

Drinking water treatment was the main barrier protecting humans from waterborne pathogens. Chlorine has been perceived as one of the most widely used and cost-effective disinfectants for deactivating microorganisms and, furthermore, guaranteeing the lingering fixation for ensured microorganism development in the water distribution network. However, *Cryptosporidium* oocysts and *Giardia* cysts could survive in water for months, and they were well-known to be resistant to chlorine disinfection.^9^ Previous studies showed that a combined treatment of UV and chlorine may be needed to eradicate *Cryptosporidium* in water treatment in a shorter duration.^10^ In addition, ozone also provided adequate inactivation of *Cryptosporidium* and *Giardia* with reasonable doses and contact times.^11^

Currently, most existing studies and routine monitoring programs focus on *Cryptosporidium* and *Giardia* in water samples of the treatment processes, which serve as indicators of source water quality and overall removal efficiency of treatment processes.^12,13^ However, protozoa in the residues generated after treatment, including sediment of the sedimentation tank and filter backwash water, remained poorly characterized. A survey of 34 drinking water treatment plants found *Cryptosporidium* levels up to 61 times higher in the initial backwash water than in the influent. Levels of *Giardia* were up to 16 times higher in the initial backwash water compared with the corresponding influent levels.^14^ Karanis et al. reported that *Giardia* cysts (42/50) and *Cryptosporidium* oocysts (41/50) were detected in samples of sand filter backwash water from a river-source drinking water plant. This contaminated water was returned to the raw water supply without being completely free of cysts and oocysts.^15^ If not managed properly, these concentrated protozoa could constitute secondary contamination sources, posing significant operational and health risks.^16^ Therefore, except for influent and effluent, some potential risk points of treatment residues require more attention.

In addition to the enumeration of *Cryptosporidium* and *Giardia*, knowledge of more kinds of waterborne protozoa and their human infection risk along drinking water treatment processes was scarce. Protozoa such as *Echinococcus ameba*, *Microsporidium*, *Enterocytozoon bieneusi*, *Toxoplasma gondii*, and *Schistosoma* have also been linked to waterborne diseases;^17,18^ screening of diverse protozoa in the drinking water treatment plant could expand the monitoring targets and provide critical information on health risk assessment and technical optimization. The human infection risk of protozoa could be confirmed by species and genotype identification. For example, at least 44 *Cryptosporidium* species and 120 genotypes have been recognized currently,^19^ while *C. hominis* and *C. parvum* accounted for 95% of human infections.^20^ *C. parvum* subtype IIc appeared to be anthropologically transmitted, whereas subtypes IIa and IId were zoonotically transmitted and are mostly found in cattle, sheep, and goats.^21^

In this study, a molecular investigation on the potential risk points of waterborne protozoa was conducted in a representative full-scale drinking water treatment plant in Shanghai, China. This plant adopted typical conventional (coagulation, sedimentation, sand filtration, and disinfection) and advanced (ozonation and activated carbon filtration) treatment processes. Except for the source water of the reservoir, influent, and effluent, three potential risk points along the treatment processes, including sediment of the sedimentation tank, backwash water of the sand filter, and backwash water of the activated carbon filter, were also considered. By integrating full-scale monitoring with advanced molecular approaches, the specific objectives of this study were (1) to screen the occurrence of waterborne protozoa using high-throughput quantitative PCR targeting 19 known protozoa and immunofluorescence assay targeting *Cryptosporidium* and *Giardia* and (2) to profile the genotypes and potential human risks of dominant protozoa using nested PCR and phylogenetic analysis. This study will provide a comprehensive understanding and optimization of techniques on the biosafety of protozoa in drinking water treatment plants.

## Materials and Methods

### Sample Collection and Processing

To investigate the occurrence of waterborne protozoa along drinking water treatment processes, a full-scale drinking water treatment plant in Shanghai, China, using the Yangtze River as a water source, was selected for sampling. Its main treatment processes included preozonation, coagulation sedimentation, sand filtration, activated carbon filtration, and disinfection, which combined conventional and advanced treatments. The advanced treatment could improve the efficiency of removing microorganisms. The supply capacity of this treatment plant was 400,000 tons per day. The run cycles for the sand filter and activated carbon filter were 52 and 168 h, respectively. The hydraulic retention times (HRTs) were approximately 4 min for preozonation, 130 min for the sedimentation tank, 12 min for the postozonation contact tank, and 30 min for the disinfection contact tank.

In October 2023, December 2023, and March 2024, reservoir, influent, effluent, backwash water, and sediment samples were collected across three distinct seasons to minimize random variation. Concretely, as shown in Figure 1, water samples were collected at the reservoir (W1), influent (W2), and effluent (W3) of the plant; in addition, to investigate the potential risk points along treatment processes, the sediment of the sedimentation tank (S1), backwash water of the sand filter (S2), and backwash water of the activated carbon filter (S3) were collected. Protozoa were concentrated from water samples of the reservoir (10L), influent (10L), and effluent (50L) in triplicate for subsequent DNA extraction and immunofluorescent assay. The sediment of the sedimentation tank (2L), backwash water of the sand filter (2L), and backwash water of the activated carbon filter (2L) were centrifuged, and the solid was used for DNA extraction in triplicate. No significant rainfall events occurred during the week preceding any sampling. A total of 18 samples were collected, stored at 4 °C in the dark, and transported to the laboratory. The physicochemical information on the samples is provided in Table S1 in Supporting Information.

**Figure 1:**
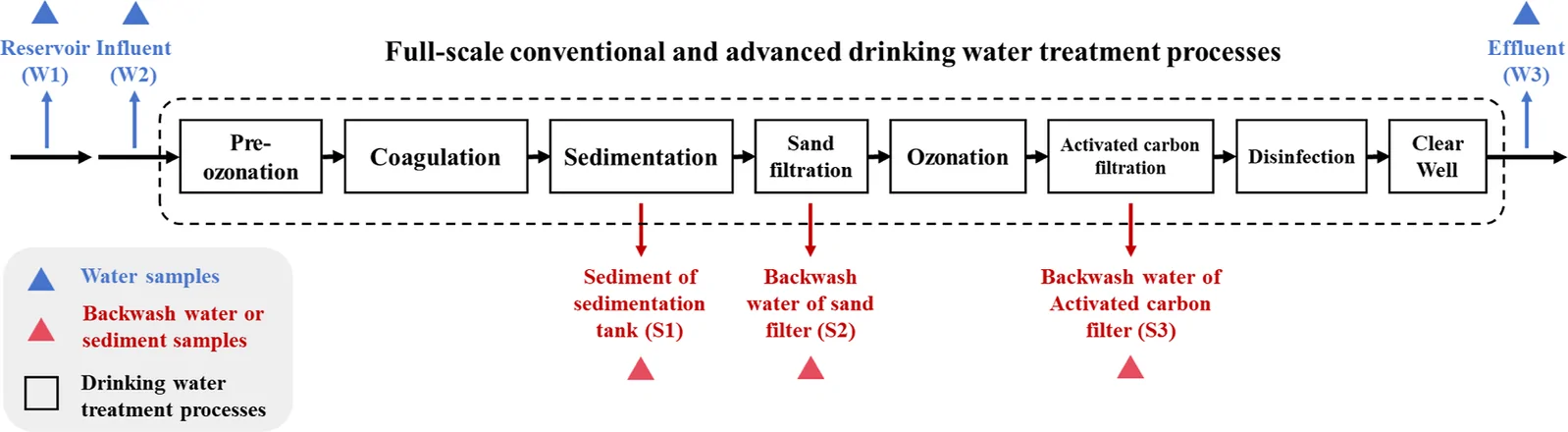
Schematic diagram of treatment processes and sampling points in a drinking water treatment plant. W1, reservoir water; W2, influent; W3, effluent; S1, sediment of the sedimentation tank; S2, backwash water of the sand filter; S3, backwash water of the activated carbon filter.

### DNA Extraction and High-Throughput Quantitative PCR (HT-qPCR) Assay

The calcium carbonate flocculation method employed complies with the Standard Examination Methods for Drinking Water (GB/T 5750.12). Water sample concentration typically uses 10 L as the standard volume. In this study, reservoir water and plant influent were concentrated at 10L. Considering that protozoan counts in effluent are generally low, the effluent concentration volume was increased to 50 L to enhance detection sensitivity. This was the same as the immunofluorescence assay, and the resulting concentrate was processed for DNA extraction. Sediment and backwash water samples were concentrated by using centrifugation. Environmental DNA was extracted from 0.5 g of the sample concentrate using the Fast DNA SPIN Kit for soil and stored at −20 °C until use.

In our previous research, a HT-qPCR method targeting 19 protozoa and three helminths, which covered a broad spectrum of waterborne protozoa and helminths, has been established.^22^ These taxa were chosen based on their waterborne characteristics and potential environmental dissemination risk according to the WHO Water Quality Guidelines (fourth edition) (Table S2). The HT-qPCR assay was conducted on OpenArray chips using an Applied Biosystems QuantStudio 12K Flex instrument (Thermo Fisher, USA), which enabled simultaneous operation of up to four OpenArray chips, allowing testing of up to 192 samples over a 3 h period. 18S rRNA and 16S rRNA genes served as quality controls. DNA was diluted to approximately 20 ng/μL, and 400 ng/μL bovine serum albumin (BSA) was added to each qPCR system to improve the amplification efficiency and mitigate potential inhibition. The detection limit of this method was 100 copies/μL DNA.^22^ In this study, samples were loaded into a 96-well plate, adding 2.5 μL of DNA sample and 2.5 μL of OpenArray Real-Time PCR Master Mix to each sample well. The standard curves exhibited *R* ^2^ values ranging from 0.990 to 0.998, with amplification efficiencies between 75% and 89% (Table S3). Positive results are shown in Table 1, and only results above the limit of quantitation are shown in Figure 2.

**Table 1:** Detection Rate (%) of Waterborne Protozoa along Full-Scale Conventional and Advanced Drinking Water Treatment Processes by HT-qPCR^a^

sampling locations	Acanthamoeba genus	Acanthamoeba culbertsoni	Acanthamoeba polyphaga	Cyclospora cayetanensis	Cryptosporidium spp.	Cryptosporidium parvum	Cryptosporidium meleagridis	Enterocytozoon bieneusi	Entamoeba histolytica	Encephalitozoon	Giardia spp.
W1	-	-	33.3%	-	-	-	-	66.7%	-	33.3%	-
W2	-	33.3%	-	-	-	-	-	33.3%	-	-	-
W3	-	-	-	-	-	-	-	-	-	-	-
S1	33.3%	-	-	-	33.3%	-	-	33.3%	33.3%	-	33.3%
S2	66.7%	33.3%	33.3%	33.3%	66.7%	33.3%	33.3%	-	-	33.3%	-
S3	-	-	-	-	33.3%	-	-	-	-	-	-
average	16.7%	11.1%	11.1%	5.6%	22.2%	5.6%	5.6%	22.2%	5.6%	11.1%	5.6%

a W1, reservoir water; W2, influent; W3, effluent; S1, sediment of the sedimentation tank; S2, backwash water of the sand filter; S3, backwash water of the activated carbon filter; -, undetectable. Only 11 positive targets were shown.

**Figure 2:**
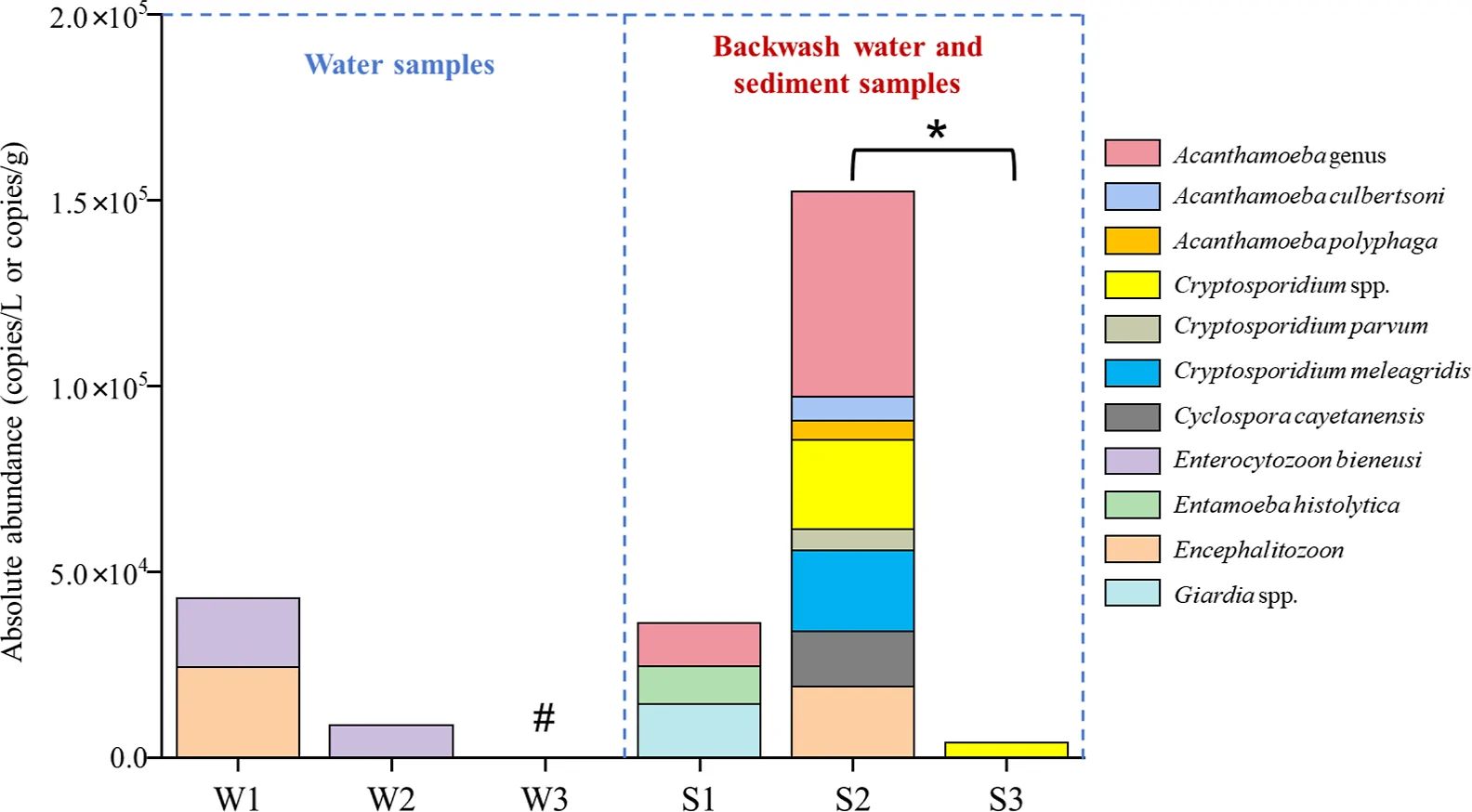
Absolute abundance of waterborne protozoa along full-scale conventional and advanced drinking water treatment processes by HT-qPCR. W1, reservoir water; W2, influent; W3, effluent; S1, sediment of the sedimentation tank; S2, backwash water of the sand filter; S3, backwash water of the activated carbon filter. # indicates below the detection limit under 10 L of water. * indicates *P* < 0.05 of the Mann–Whitney U test between two groups.

### Detection of *Cryptosporidium* and *Giardia* by an Immunofluorescent Assay (IFA)

The morphological examination and enumeration of *Cryptosporidium* oocysts and *Giardia* cysts were performed for reservoir, influent, and effluent samples using previously described methods involving flocculation, flotation, labeling with a monoclonal antibody, and microscopic analysis. Quality control tests, in which 10 L of distilled water was seeded with a known number of (oo)­cysts, revealed that the method permitted an average recovery rate of 32.0% for *Cryptosporidium* oocysts and 28.0% for *Giardia* cysts, which met the acceptance criteria (more than 20%) of the Standard Examination Methods for Drinking Water (GB/T 5750.12-2023). More details are shown in Supporting Information.

### Genotyping of *Cryptosporidium* and *E. bieneusi* by Nested PCR

For species identification of *Cryptosporidium*, an approximately 840 bp fragment of the small-subunit (SSU) rRNA gene was amplified by nested PCR as previously described.^23,24^ *Cryptosporidium* species were differentiated by a DNA sequence analysis. To further identify the genotypes of *C. parvum*, nested PCR and sequence analyses of a 400 bp fragment of the 60 kDa glycoprotein (*gp60*) gene were performed.^25^ Genotyping of *E. bieneusi* was conducted by nested PCR of a 392 bp fragment of the ITS gene as previously described.^25^ Detailed primer information, PCR reaction system, and conditions were listed in Supporting Information (Tables S4–S12). Three parallel PCR amplification experiments were performed for each sample, and positive and negative controls were set. The PCR products were sent to Sangong Biotech (Shanghai) Co., Ltd. for Sanger sequencing.

### Phylogenetic and Statistical Analysis

Relationship of genotypes of *C. parvum* and *E. bieneusi* identified in this study and known genotypes published previously were inferred using a neighbor-joining estimation using MEGA12.0. Visualization was conducted using Origin Pro software (v. 2022, Origin Lab Corporation, USA). The Mann–Whitney U test was a nonparametric method which does not demand the normal distribution in data. It was used to compare the difference of absolute abundance of waterborne protozoa along full-scale conventional and advanced drinking water treatment processes by HT-qPCR, and the sample size for each group was three replicates.

## Results and Discussion

### Screening of Waterborne Protozoa along Drinking Water Treatment Processes Using High-Throughput Quantitative PCR (HT-qPCR)

Among the HT-qPCR targets of 19 protozoa and three helminths, a total of 11 protozoan targets were detectable during the treatment processes in the drinking water treatment plant. *Cryptosporidium* spp. (22.2%) and *E. bieneusi* (22.2%) exhibited the highest detection rates, followed by the *Acanthamoeba* genus (16.7%), *Acanthamoeba culbertsoni* (11.1%), *Acanthamoeba polyphaga* (11.1%), and *Encephalitozoon* (11.1%) (Table 1). The occurrence of protozoa varied significantly across the treatment processes. Three and two targets were detectable in reservoir and influent samples, respectively, while no positive results were obtained in the effluent. Five, eight, and one targets were detectable in the sediment of the sedimentation tank, backwash water of the sand filter, and backwash water of the activated carbon filter, respectively (Table 1).

In reservoir samples, *E. bieneusi* showed the highest detection rate (66.7%) with concentrations ranging from ND to 4.5 × 10^4^ copies/L, followed by *A. polyphaga* (33.3%, ND-1.2 × 10^3^ copies/L) and *Encephalitozoon* spp. (33.3%, ND-7.2 × 10^4^ copies/L). In the influent, *E. bieneusi* (33.3%, ND-2.6 × 10^4^ copies/L) was found. In the sediment of the sedimentation tank, the *Acanthamoeba* genus (33.3%, ND-1.2 × 10^4^ copies/g), *Entamoeba histolytica* (33.3%, ND-3.1 × 10^4^ copies/g), and *Giardia* spp. (33.3%, ND-4.2 × 10^4^ copies/g) were found. In the backwash water of the sand filter, *Cryptosporidium* spp. showed a 66.7% detection rate (ND-2.7 × 10^4^ copies/g), followed by *Acanthamoeba* spp. (66.7%, ND-1.7 × 10^5^ copies/g), *A. culbertsoni* (33.3%, ND-2.0 × 10^4^ copies/g), *A. polyphaga* (33.3%, ND-1.6 × 10^4^ copies/g), *C. parvum* (33.3%, ND-1.7 × 10^4^ copies/g), *Cryptosporidium meleagridis* (33.3%, ND-6.5 × 10^4^ copies/g), *Cyclospora cayetanensis* (33.3%, ND-4.5 × 10^4^ copies/g), and *Encephalitozoon* spp. (33.3%, ND-5.8 × 10^4^ copies/g). In the backwash water of the activated carbon filter, *Cryptosporidium* spp. (33.3%, ND-1.1 × 10^4^ copies/g), was detectable. The specific detection results of HT-qPCR were summarized in Supporting Information (Table S13).

Using HT-qPCR, the average abundance with the unit of copies/L or copies/g among different samples was quantified (Figure 2). Protozoa abundance of reservoir and influent samples was 4.3 × 10^4^ copies/L and 8.5 × 10^3^ copies/L, respectively. Protozoan abundance in backwash water and sediment samples was higher than that in reservoir and influent samples. Backwash water of the sand filter showed the highest abundance of 1.5 × 10^5^ copies/g. In the sediment of the sedimentation tank and backwash water of the activated carbon filter, protozoan abundance was 3.6 × 10^4^ copies/g and 3.8 × 10^3^ copies/g, respectively. To the best of our knowledge, a few studies reported waterborne protozoa, except for *Cryptosporidium* and *Giardia*. In a year-long study of surface water protozoa near Paris, *E. bieneusi* was found in only two river samples.^26^ Previous high-throughput qPCR screening results of drinking water samples from the Yangtze and Yellow Rivers (*n* = 46) showed detection rates of 54.3% for the *Acanthamoeba* genus (7.5 × 10^4^ copies/L), 8.7% for *Cryptosporidium* spp. (3.5 × 10^3^ copies/L), and 8.7% for *E. bieneusi* (3.5 × 10^3^ copies/L),^22^ which exhibited similar detection rates and abundance to this study.

Multiple drinking water treatment processes contributed to protozoa removal, and the removal efficiency for protozoa is commonly characterized by the log reduction value (LRV). Conventional coagulation and sedimentation mainly reduced turbidity and natural organic matter; at the same time, pathogens inevitably became adsorbed to these coagulated particles and settled out and thus achieved the average LRV of 1.1 (95% CI 0.8–1.3) for protozoa.^27^ In addition, dissolved air flotation was also useful for removing *Cryptosporidium* and *Giardia* as they gain buoyancy when their flocs attach to rising bubbles.^28^ During media filtration, microorganisms were removed by adsorption, interception, or sedimentation onto the granular media, and coagulation-induced microbe-associated particles were the key factor in intercepting pathogens from the water. It was reported that the average LRV of protozoa by granular media filtration was 3.0 (95% CI 2.8–3.3).^27^ Granular activated carbon (GAC) filtration can achieve an average LRV of 1.5 (95% CI 0.9–2.1) for protozoa.^27^ In this study, diverse and abundant protozoa were found in the backwash water of the sand filter, which also indicated that media filtration was the key step in intercepting protozoa. Thus, while no protozoa were detected in the effluent, their presence in process residuals—particularly sand filter backwash water—highlights these points as potential risks requiring attention.

Currently, *Cryptosporidium* and *Giardia* are the main research targets in drinking water protozoa, and information about a broad spectrum of waterborne protozoa in drinking water is still scarce. This study employed HT-qPCR methods to screen 19 waterborne protozoan targets throughout the drinking water treatment processes. Except for *Cryptosporidium* and *Giardia*, we highlighted that *E. bieneusi* also needs attention when considering abundance and detection frequencies (Table 1, Figure 2). *E. bieneusi* is the most diagnosed species in humans; it is responsible for opportunistic infections in AIDS patients and other immunocompromised patients such as organ transplant recipients or cancer patients, as well as travelers, children, and the elderly.^29^ In 2013, an increased occurrence of *E. bieneusi* was reported in a pig carcass incident in the Huangpu River.^30^ Between June and September 1995, the incidence of *E. bieneusi* in Lyon, France, increased 10-fold. Statistical analysis revealed an association between patients’ residential areas and infection rates, supporting a hypothesis of waterborne transmission. Notably, the affected residential zones were all supplied by the same drinking water distribution subnetworks. In addition, *Acanthamoeba*, *Encephalitozoon*, *A. polyphaga*, and *A. culbertsoni* were also identified in this study. Therefore, a high-throughput cost-effective solution for screening waterborne protozoa in drinking water sources and treatment plants was recommended.

### Enumeration of *Cryptosporidium* and *Giardia* by an Immunofluorescent Assay

Table 2 shows the count of *Cryptosporidium* and *Giardia* in reservoir, influent, and effluent samples, and the detection rate was further calculated. The concentration range was 0–4 oocysts/10L for *Cryptosporidium* and 0–3 cysts/10L for *Giardia*. The detection rates of *Cryptosporidium* and *Giardia* in the reservoir water and influent reached 66.7%, while the result in the effluent was zero. Immunofluorescence analysis images of *Cryptosporidium* oocysts and *Giardia* cysts are shown in Figure S1.

**Table 2:** Counting of *Cryptosporidium* and *Giardia* Using an Immunofluorescent Assay (IFA)

sampling time	Oct. 2023		Dec. 2023		March 2024		detection rate	
	Cryptosporidium	Giardia	Cryptosporidium	Giardia	Cryptosporidium	Giardia	Cryptosporidium	Giardia
reservoir (counts/10L)	2	2	0	1	2	0	66.7%	66.7%
influent (counts/10L)	4	3	2	0	0	1	66.7%	66.7%
effluent (counts/50L)	0	0	0	0	0	0	0	0

Numerous studies have reported the enumeration of *Cryptosporidium* and *Giardia* in drinking water sources using an immunofluorescent assay. In an investigation of *Cryptosporidium* contamination in Shanghai, Zhang et al. reported detection rates of 8.0% for *Cryptosporidium* and 12% for *Giardia* in the Huangpu River, respectively.^31^ Moreno-Mesonero et al. reported detection rates of 50.0% for *Cryptosporidium* and 65.0% for *Giardia* in the influent of a drinking water treatment plant.^12^ This indicated the prevalence of contamination by these protozoa in drinking water treatment plants. The occurrence of *Cryptosporidium* and *Giardia* at different time periods indicates that the contamination may exhibit an intermittent pattern yet still poses a potential health risk. In warm and wet locations, expected precipitation can serve as a reliable predictor for the incidence of cryptosporidiosis. Extreme meteorological events, such as heavy rainfall, droughts, and heat waves, may substantially alter a seasonal pattern in disease incidence.^32^ Therefore, enhanced monitoring of source water and stricter control of pollution sources are required to prevent sudden increases in pathogen loads.

### Genotyping of the Two Predominant Protozoan Species

In this study, *Cryptosporidium* or *E. bieneusi* showed frequent occurrence compared to other protozoa (Table 1), and thus, they were selected for further genotype identification.

#### Genotyping of *C. parvum*

The DNA of all samples was used for species identification using nested PCR and sequencing of the small-subunit (SSU) rRNA gene, and only *C. parvum* was found in the reservoir water, influent, sediment of the sedimentation tank, and backwash water of the activated carbon filter. An amplicon of the *gp60* gene by nested PCR was then conducted for positive samples, and results were analyzed according to the phylogenetic relationship between sequences in this study and other genotypes previously deposited in GenBank (Figure 3). The *C. parvum* in this study was identified as subtype IIdA19G1, which belonged to the IId group and has been found in mammals such as cattle, rodents, calves, and humans.^33^ (Figure 3). Human infection cases caused by subtype IIdA19G1 have been previously reported.^34,35^ Mammalian feces are recognized as a major source of parasite introduction into surface waters.^36^ Previous studies have shown that IIdA19G1 is the predominant *C. parvum* subtype in dairy cattle in Heilongjiang Province, China. In a 2016 outbreak on a farm in Jiangsu Province, approximately 360 newborn calves died of diarrhea. In this outbreak, among four *Cryptosporidium* species identified, *C. parvum* accounted for about 49.1% of infections, and all *C. parvum* were classified as IIdA19G1.^37^ It is noteworthy that the IIdA19G1 subtype has also been detected in stool samples from HIV-positive patients.^38^ The broad host range and strong transmission capacity of the IIdA19G1 subtype pose significant challenges for cryptosporidiosis prevention and control, highlighting the need for focused attention to its public health risk.

**Figure 3:**
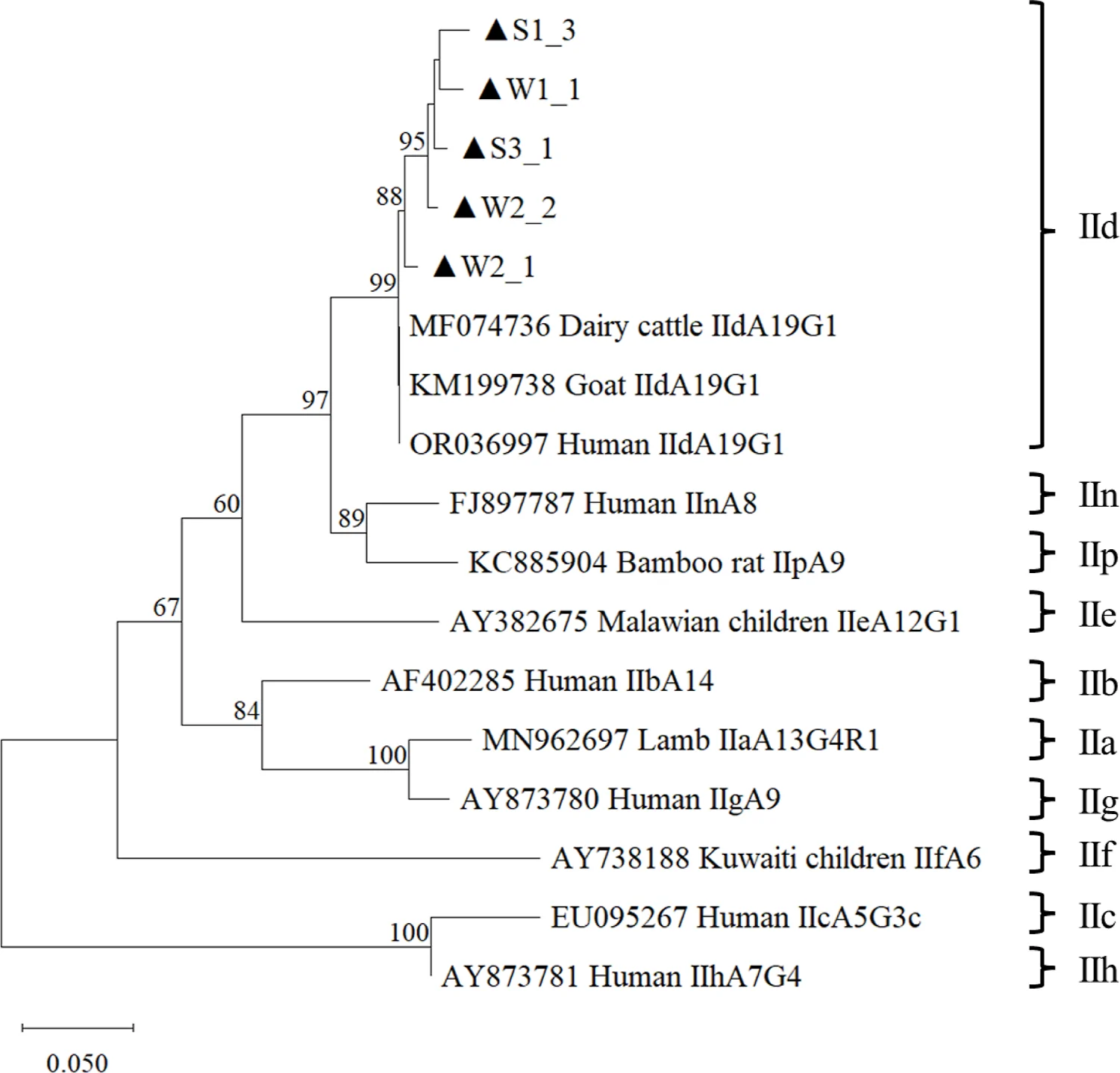
Phylogenetic relationship of *Cryptosporidium parvum* genotypes identified in this study and other genotypes previously deposited in GenBank as inferred by a neighbor-joining analysis of the 60 kDa glycoprotein (*gp60*) gene. Bootstrap values greater than 50% from 1000 replicates are shown on nodes. Samples of this study were indicated by “▲”. W1, reservoir water; W2, influent; S1, sediment of the sedimentation tank; S3, backwash water of the activated carbon filter.

#### Genotyping of *E. bieneusi*

Globally, nearly 500 genotypes of *E. bieneusi* have been recognized, with hosts ranging from humans, livestock, and companion animals to wild mammals, birds, and aquatic environments.^29^ Recently, *E. bieneusi* was classified as fungi,^39^ and thus, it was viewed as a fungus-like protist parasite.^40^ The new version of the glossary of parasitic diseases issued by the China National Center for Disease Control and Prevention (WS/T 10043-2025) noted that its biological classification remains controversial; it is classified as a microsporidian, but its morphology and biological characteristics place it between protozoa and fungi. And it is still classified as a protozoan in the WHO Water Quality Guidelines (fourth edition). *E. bieneusi* is the most significant zoonotic microsporidian species worldwide and is primarily transmitted via the fecal-oral route.^41^ Sequence analysis of the nested PCR products of the ITS gene exhibited only one genotype of *E. bieneusi*, AAE1, which was detected in the influent, sediment of the sedimentation tank, backwash water of the sand filter, and backwash water of the activated carbon filter (Figure 4). In 2023, Baz-González et al. first identified two *E. bieneusi* genotypes from fecal samples of *Atelerix algirus*, designating them AAE1 and AAE2, clustering within Group 1.^42^ Furthermore, genotype AAE1 has also been detected in *Rattus rattus*.^43^ Until now, human pathogenicity of AAE1 was still unknown, but AAE1 belonged to *E. bieneusi* Group 1 and Group 2, which exhibited broad host adaptability and represented the major zoonotic types.^29^

**Figure 4:**
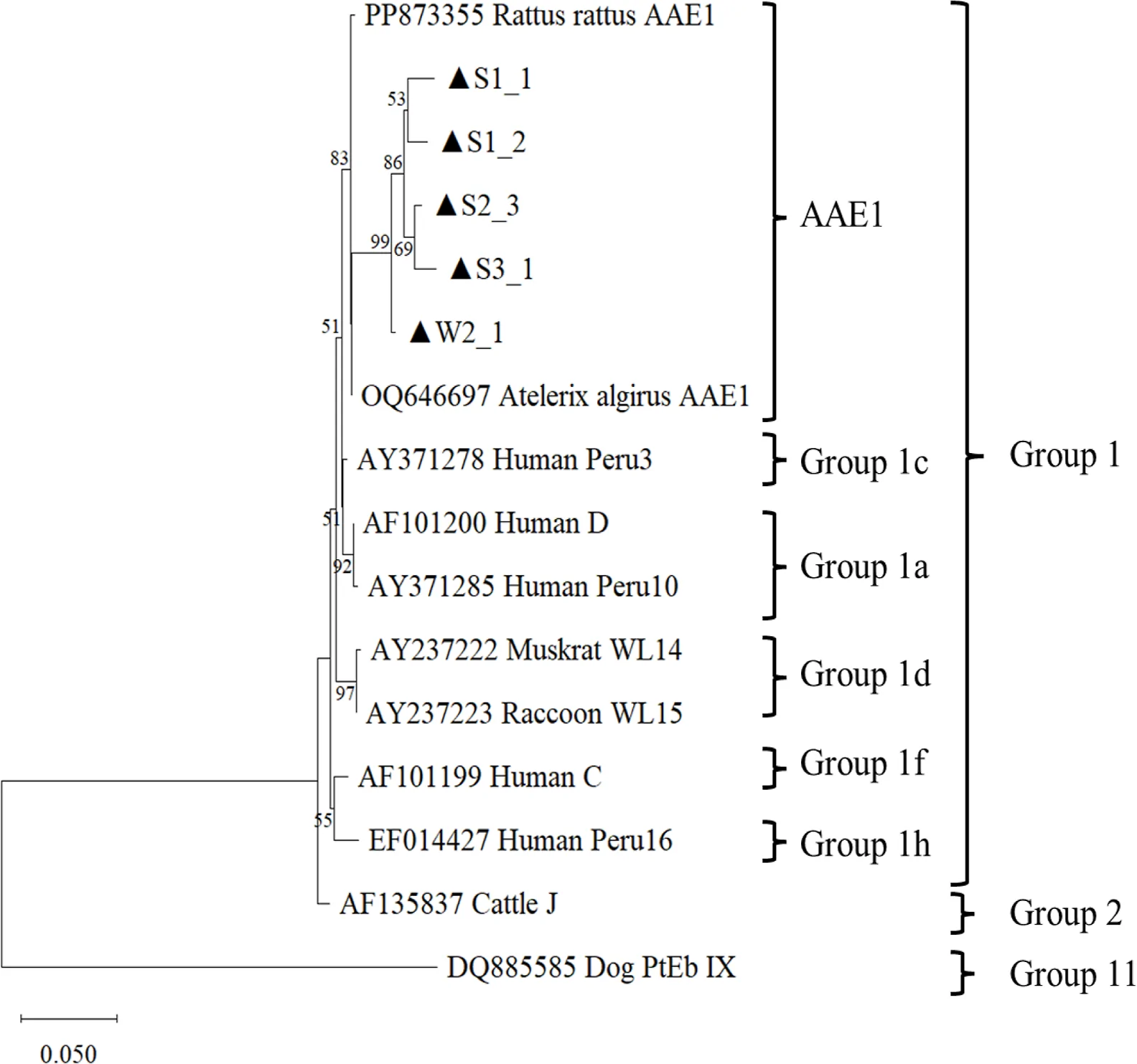
Phylogenetic relationship of *Enterocytozoon bieneusi* genotypes identified in this study and other genotypes previously deposited in GenBank as inferred by a neighbor-joining analysis of the ITS gene. Bootstrap values greater than 50% from 1000 replicates are shown on nodes. Samples of this study were indicated by “▲”. W2, influent; S1, sediment of the sedimentation tank; S2, backwash water of the sand filter; S3, backwash water of the activated carbon filter.

### Recommendations for Health Risk Management and Control

#### Inactivation of Protozoa in Drinking Water Treatment Plants

Chlorine disinfection is the most commonly used disinfection method in drinking water treatment plants; however, many protozoa, including *Acanthamoeba*, *Cryptosporidium*, and *Giardia*, exhibit varying degrees of resistance to chlorine disinfection.^10,44–46^ Qian et al. reported that UV irradiation usually inactivated *Cryptosporidium* by attacking the nucleic acid and thereby preventing multiplication of the parasite.^47^ Owing to its oxidizing properties, ozone is currently known as one of the most efficient microbicides and could break the cell membrane or protoplasm, making it impossible to activate bacteria, virus, and protozoa cells.^48^ If necessary, advanced treatment processes such as ozonation are necessary for protozoan removal in drinking water treatment plants.^49^ In addition to conventional treatment, the drinking water treatment plant in this study employed ozone-activated carbon advanced treatment, which contributed to the effective removal of waterborne protozoa in the effluent. This provided insight into the optimization of treatment processes to ensure drinking water biosafety.

#### Additional Treatment on Backwash Water and Sediment

The backwash water of the sand filter served as an enrichment zone for protozoa, and it commonly returns to the influent. Thus, it caused a potential risk of protozoa accumulation. Additional treatment of backwash water was necessary for decreasing protozoan loading in drinking water treatment plants. Previous studies have confirmed that treatment of backwash water through coagulation followed by clarification effectively removed *Cryptosporidium* oocysts,^50^ and ozone (with contact times of 5 and 10 min at doses of 10 mg O_3_ per L and 7.5 mg O_3_ per L, respectively) can be an effective method to remove the (oo)­cysts of *C. parvum* and *Giardia* in backwash water.^51^ Currently, the primary methods for the sediment of the sedimentation tank from drinking water plants include landfill disposal, incineration, or systematic discharge into wastewater treatment plants.^52^ Other common approaches include the discharging of the sediment of drinking water treatment plants into rivers, which still occurs in many undeveloped countries.^53^ Harmless treatment of the sediment together with health risk assessments was still needed in future studies. In this study, we identified potential risks from *Cryptosporidium*, *Giardia*, *E. bieneusi*, and *Acanthamoeba* et al. in backwash water and sediment. This indicates the necessity for additional measures like ozone or UV for these treatment residues along with conducting health risk assessments.

#### Monitoring, Risk Assessment, and Source Tracking

HT-qPCR technology adopted in this study, with its high-throughput characteristics and multitarget detection capabilities, is an ideal method for monitoring protozoa in drinking water treatment plants.^22^ Other frequently detected protozoa, such as *E. bieneusi* and *Acanthamoeba*, in addition to *Giardia* and *Cryptosporidium*, were incorporated into the monitoring systems at drinking water plants to comprehensively assess the distribution of protozoa. It is necessary to conduct quantitative microbial risk assessments (QMRAs) for protozoa frequently detected in drinking water plants.^54^ Furthermore, the species and genotype identification of protozoa should be performed using molecular methods to facilitate the assessment of human infection risk. Most pathogenic protozoa are derived from human and animal fecal matter.^55–57^ Tracing the source of protozoan contamination helps block protozoan risks at their origin. Investigations should be conducted on wildlife habitats around the water source area and their fecal contamination pathways. A geographical information system (GIS) can prepare a geographical pattern of disease distribution and help to develop early warning systems for early detection of infectious diseases.^58^ The integration of molecular analysis with advanced technologies such as GIS can provide valuable information within the GIS framework to accurately quantify the spatial distribution of aquatic biota and water chemistry and thus determine the risks to water bodies.^59^ Implementing this framework will enhance the predictive capability and support evidence-based decision-making. It is noted that the sampling frequency and detection precision of this study should be enhanced, which requires further studies.

## Conclusions

In conclusion, this study employed an integrated approach combining high-throughput qPCR, nested PCR, and immunofluorescent assay to conduct a systematic assessment of protozoa in a full-scale drinking water treatment plant. Eleven protozoa targets were detectable, with *Cryptosporidium* spp. and *E. bieneusi* exhibiting the highest detection rates (22.2%). While no protozoa were detected in the effluent, the backwash water of the sand filter was identified as a critical risk point harboring the highest diversity of protozoa. The immunofluorescence assay found the presence of *Cryptosporidium* (66.7%) and *Giardia* (66.7%) in the source water and influent, but none in the effluent. Genotyping characterized the dominant *Cryptosporidium* as the zoonotic subtype *C. parvum* IIdA19G1, while the identified *E. bieneusi* genotype AAE1 (Group 1) still has no evidence of human infection. These findings highlighted that although the protozoa in effluent were undetectable, backwash water and sediment during treatment processes were identified as potential risk points. This required further sampling and seasonal monitoring, risk assessment, and optimization of additional treatment for backwash water and sediment.

## Supplementary Material



## Notes

Additional experimental details, materials, and methods, including photographs of the experimental setup (PDF)
