# Supplementary material for: Occurrence of Waterborne Protozoa in a Full-Scale Drinking Water Treatment Plant in China by High-Throughput Screening and Genotyping
Source: Environ Health (Wash). 2026 May 6;4(9):1977–86. doi: 10.1021/envhealth.5c00863 (PMC13595302; doi:10.1021/envhealth.5c00863)
Supplement: Supplementary file 1 [file eh5c00863_si_001.pdf]

Supporting information

Occurrence of waterborne protozoa in a full-scale drinking water treatment plant in China by high-throughput screening and genotyping

Huican Zhang[1,2]#, Ziming Han[2]#, Fuming Duan[2], Shumin Xiao[3], Min Yang[4,5], Yu Zhang[2,5]*

1 Henan Institute of Advanced Technology, Zhengzhou University, Zhengzhou 450003, China
2 Key Laboratory of Environmental Aquatic Chemistry, State Key Laboratory of Regional Environment and Sustainability, Research Center for Eco-Environmental Sciences, Chinese Academy of Sciences, Beijing 100085, China
3 School of Environmental and Municipal Engineering, Tianjin Chengjian University, Tianjin 300384, China
4 National Engineering Research Center of Industrial Wastewater Detoxication and Resource Recovery, Research Center for Eco-Environmental Sciences, Chinese Academy of Sciences, Beijing, 100085, China
5 University of Chinese Academy of Sciences, 100049, Beijing, China

# Co-first authors with equal contributions.

* Corresponding authors
Prof. Yu Zhang
Research Center for Eco-Environmental Sciences, Chinese Academy of Sciences, Beijing 100085, China
Tel.: 86 10 62919883; Email: zhangyu@rcees.ac.cn

Sample elution, concentration, and purification

A calcium carbonate flocculation concentration method was used in this study [1, 2].The samples were mixed thoroughly before the separate addition of 100 ml 1 mol/L $CaCl_2$ and 100 ml 1 mol/L $NaHCO_3$. After further mixing the pH of samples was adjusted to 10 by the addition of 10 mol/L NaOH. The samples were left at room temperature for 14 h and the supernatant fluid was removed by vacuum aspiration. Care was taken not to disturb the calcium carbonate residue. The residue was dissolved by adding 200 ml of 10% w/v sulphamic acid and shaking vigorously for 15 s before decanting into centrifuge bottles. The barrels were then rinsed with 200 ml of 0.01% v/v solution of Tween 80 and shaken vigorously. The fluid was again decanted and the process repeated with a further 100 ml of Tween 80 solution. After centrifuging at 3000 g for 10 min the supernatant fluids were removed leaving 40-50 ml. The pellets were then resuspended and transferred to a 50 ml centrifuge tube. Samples were centrifuged again at 3000 g and the supernatant fluid removed. The original centrifuge tube was then rinsed with 50 ml of Tween 80 solution which was then used to resuspend the pellet.

The concentrated oocysts were separated from other particulate materials by flotation on Percoll-sucrose gradients, as described previously [3]. Briefly, the particulate suspension (3 mL) was transferred to a 15-mL conical centrifuge tube and underlaid with approximately 5 mL of Percoll-sucrose solution (specific gravity, 1.10), using a capillary pipette. The Percoll-sucrose gradients were centrifuged at 20 C in a swinging bucket rotor at 1,050×*g* for 10 min. The entire fluid layer above the pellet in each flotation tube was harvested by pipette and transferred into a fresh 50-mL conical centrifuge tube. To avoid losses that might occur, the pellet was resuspended in 3 mL of PBST, repurified, harvested, and transferred into the same conical centrifuge tube, and the volumes were adjusted to 40 mL with additional PBS buffer. The harvests were pelleted by centrifugation at 1,050×*g* for 10 min at 20 C. Supernatants were aspirated off, leaving a 3-mL concentrated sample solution, including the pellets.

Sample staining, examination, and quality assurance

The method for sample staining was according to a previous study [4]. Twenty-five-millimeter diameter, 3.0-µm-pore-size cellulose acetate membrane filters (Advantec MFS, Inc. Japan), which were marked in the middle with a circle, using a DAKO pen (Dako North America, Inc. California, USA) to designate the area of inoculation, were wetted in PBS, and then, placed onto a glass filter holder. The purified sample was inoculated within the encircled area for filtration and washed twice by passing 1 mL of PBS for each. Then 0.5 mL of 1% bovine serum albumin (BSA) in PBS was applied and incubated for 1 min. The membrane filter was transferred on a fresh plane glass slide and stained with a combined fluorescein isothiocyanate (FITC) conjugated anti-*Cryptosporidium* monoclonal antibody (Waterborne, Inc., New Orleans, LA). An aliquot (100 µL) of antibody was applied to the filter and the slide was placed in a humid chamber in the dark. After incubation at room temperature for 30 min, 50 µL of

4′,6′-diamidino-2-phenylindole (DAPI) was applied and incubated for 10 min. The membrane filter was transferred back onto the filter holder, rinsed with 2 mL of PBS, and then treated sequentially with 1 mL of 30%, 70%, and 90% ethanol solutions containing 5% glycerol. Positive and negative staining controls were routinely included according to the recommendations of the manufacturer.

For microscopic observation, the membranes were mounted on glass microscope slides with 75 μL of 1,4-diazabicyclo[2.2.2]octane (DABCO)-glycerol mounting medium and sealed with coverslips and nail varnish. The entire encircled area of the membrane was systematically examined using fluorescence microscopy (Olympus BX51, USA) for the FITC, DAPI fluorescence and differential interference contrast (DIC) examination. *Cryptosporidium* spp. oocysts were identified on the basis of their size, shape, and internal structures. Only those meeting the morphological criteria of oocysts, as described in method 1623 [5], were regarded as positive and recorded. The calculated numbers were used to extrapolate to concentrations of oocysts per liter of sample.

To assure acceptable method performance, initial precision and recovery (IPR) tests were included prior to the initiation of field sampling and method blanks were included with each sampling campaign as recommended in method 1623 [5]. For the IPR tests, four replicate trials were performed, 10 L of reagent water was spiked directly with *Cryptosporidium parvum* oocyst (Iowa isolate, approximately 1 month old; supplied by Waterborne Inc., New Orleans, LA, USA), and then the seeded reagent water was further treated using the method described above.

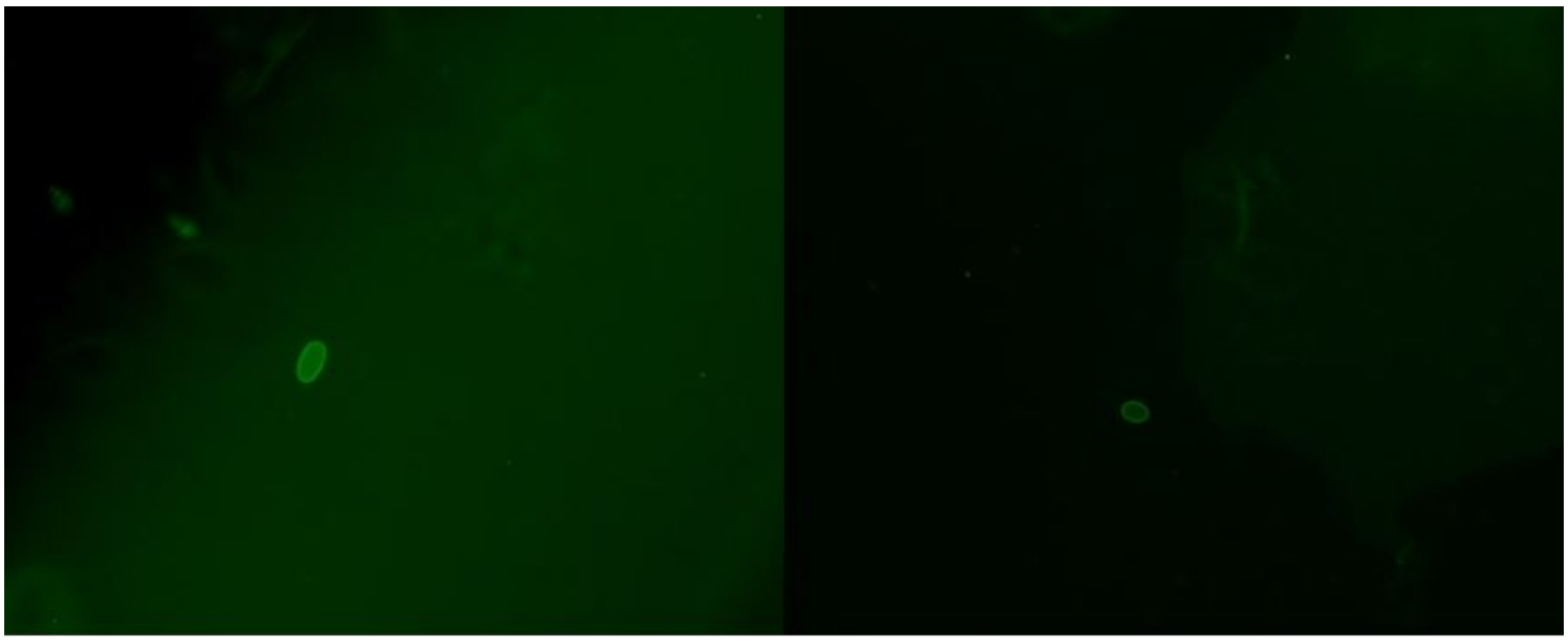

(a)

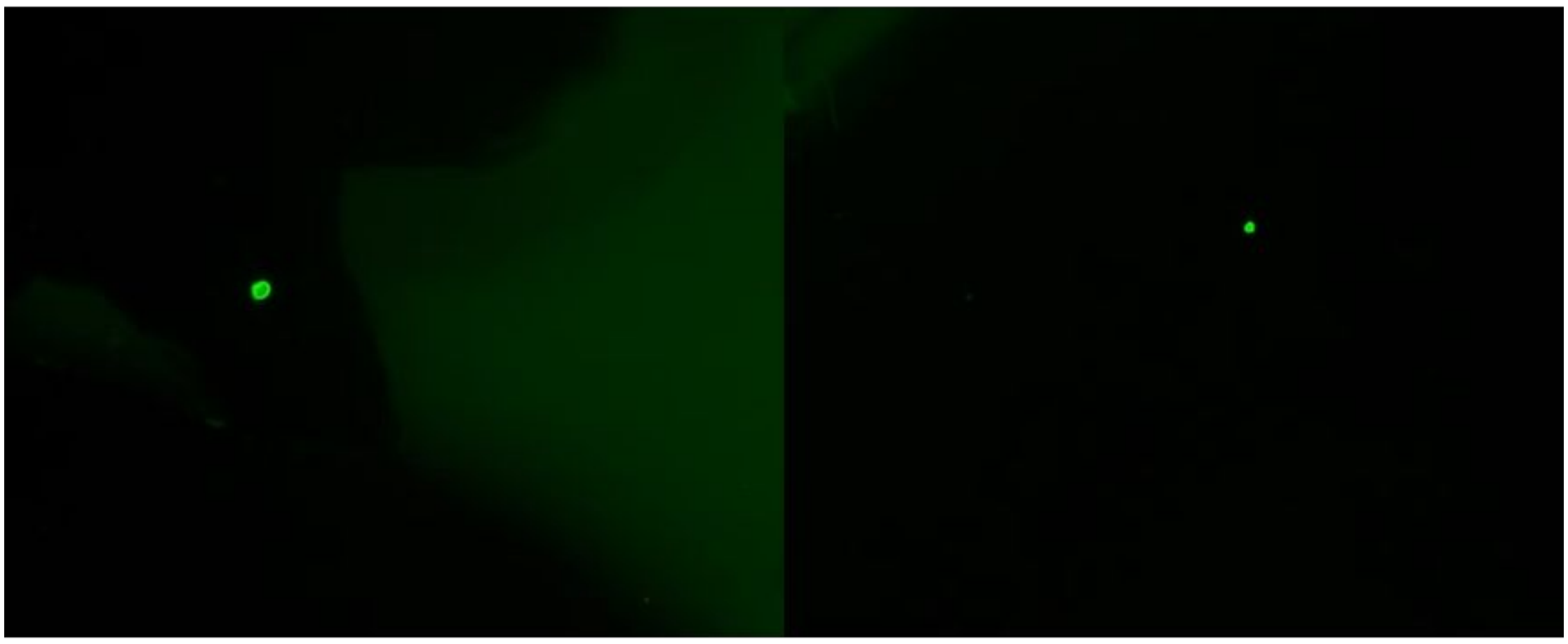

(b)

Figure S1 (a), Microphotography of *Giardia* spp. Cyst; (b), Microphotography of *Cryptosporidium* spp. oocyst

Table S1 physicochemical information of the samples

2023.10	Reservoir	Influent	Sedimentation	Sand filtration	Activated carbon filtration	Disinfection	Effluent
Temperature	21.4	21.3	21.4	21.3	21.3	21.3	21.3
pH	7.93	7.97	7.68	7.77	7.42	7.29	7.28
Turbidity	20.3	21	6.06	0.21	0.188	0.04	0.01
DO	8.5	8.33	8.37	8.23	8.41	8.35	8.37
Conductivity							
Residual Chlorine	0.04	0.03	0.17	0.03	0.03	0.74	0.75
Total Chlorine	0	0.04	0.28	0.07	0.03	1.14	1.12
**2023.12**	**Reservoir**	**Influent**	**Sedimentation**	**Sand filtration**	**Activated carbon filtration**	**Disinfection**	**Effluent**
Temperature	7.03	7.84	7.39	7.32	7.41	7.2	7.33
pH	7.96	8.03	7.85	7.82	7.87	7.69	7.31
Turbidity	45.43	37.11	2.39	0.07	0.04	0.03	0.02
DO	12.63	12.57	12.12	11.49	12.61	12.06	12.22
Conductivity		354.7					
Residual Chlorine	0.01	0.01	0.01	0.01	0.02	0.78	0.82
Total Chlorine	0.02	0.03	0.16	0.03	0.03	1.02	1.03
**2024.3**	**Reservoir**	**Influent**	**Sedimentation**	**Sand filtration**	**Activated carbon filtration**	**Disinfection**	**Effluent**
Temperature	13.59	13.62	13.53	13.27	13.88	13.64	13.91
pH	7.82	7.74	7.82	7.92	7.68	7.71	7.29
Turbidity	31.52	30.34	2.29	0.034	0.034	0.029	0.028
DO	9.42	9.37	9.38	9.67	9.69	9.73	9.81
Conductivity		342.5					
Residual Chlorine	0.04	0.02	0.05	0.03	0.02	0.98	1.01
Total Chlorine	0.02	0.06	0.23	0.04	0.05	1.13	1.02

Table S2 Basic information of waterborne protozoa and helminths

Pathogen	Detection target	Health significance	Persistence in water supplies	Resistance to chlorine	Relative infectivity	Important animal source
Acanthamoeba	*Acanthamoeba*	-	-	-	-	-
	Acanthamoeba culbertsoni	High	May multiply	Low	High	No
	Acanthamoeba castellanii	High	May multiply	Low	High	No
	Acanthamoeba polyphaga	High	May multiply	Low	High	No
Balantioides	*Balantidium coli*	High	Moderate	High	High	No
Blastocystis	*Blastocystis hominis*	High	May multiply	High	Moderate	Yes
Cryptosporidium	*Cryptosporidium* spp.	-	-	-	-	-
	Cryptosporidium parvum	High	Long	High	High	Yes
	Cryptosporidium hominis	High	Long	High	High	Yes
	Cryptosporidium meleagridis	High	Long	High	High	Yes
Cyclospora	*Cyclospora cayetanensis*	High	Long	High	High	Yes
Enterocytozoon	*Enterocytozoon bieneusi*	High	Long	High	High	Yes
Entamoeba	*Entamoeba histolytica*	High	Moderate	High	High	No
Encephalitozoon	*Encephalitozoon*	High	Moderate	High	Moderate	Yes
Giardia spp.	*Giardia* spp.	-	-	-	-	-
	Giardia lamblia	High	Moderate	High	High	Yes
Naegleria	*Naegleria fowleri*	High	May multiply	Low	Moderate	No
Isospora	*Isospora belli*	Moderate	Moderate	High	High	Yes
Toxoplasma gondii	*Toxoplasma gondii*	High	Long	High	High	Yes
Echinococcus	*Echinococcus*	High	Long	Low	Moderate	Yes
Dracunculus	*Dracunculus medinensis*	Low	Moderate	Moderate	High	No
Schistosoma	*Schistosoma* spp.	High	Short	Moderate	High	Yes

Table S3 Standard curves, correlation coefficients, amplification efficiencies for detection target of HT-qPCR.

Type	No.	Target	Standard curve	Correlation coefficient(R^2)	Amplification efficiency (%)	Assay ID*
Protozoa	1	*Acanthamoeba*	y=-4.02364x+41.47677	0.991	77	AP7DU7W
	2	*Acanthamoeba culbertsoni*	y=-3.63068x+38.9712	0.994	89	APEPZZ9
	3	*Acanthamoeba castellanii*	y=-3.79737x+40.87273	0.995	83	APDJ7GC
	4	*Acanthamoeba polyphaga*	y=-3.62098x+39.56447	0.993	89	APAAJCZ
	5	*Balantidium coli*	y=-4.00778x+41.20159	0.996	78	AP2XA9F
	6	*Blastocystis hominis*	y=-3.9753x+40.84028	0.995	78	APGZFRJ
	7	*Cryptosporidium hominis*	y=-3.75584x+39.65748	0.997	85	APAAJDD
	8	*Cryptosporidium meleagridis*	y=-4.1254x+40.80545	0.997	75	AP9HTZD
	9	*Cryptosporidium* spp.	y=-3.96503x+39.27098	0.992	79	Pr07922253_s1
	10	*Cryptosporidium parvum*	y=-3.64984x+38.73503	0.998	88	APGZGDP
	11	*Cyclospora cayetanensis*	y=-3.73214x+38.48602	0.992	85	Pr07922173_s1
	12	*Encephalitozoon*	y=-3.7154x+39.86931	0.995	86	APDJ7HX
	13	*Entamoeba histolytica*	y=-3.71967x+38.82379	0.998	86	AP2XCMY
	14	*Enterocytozoon bieneusi*	y=-3.71797x+39.01301	0.993	86	AP7DWCR
	15	*Giardia* spp.	y=-3.71548x+39.16803	0.995	86	AP473C7
	16	*Giardia lamblia*	y=-3.79424x+38.83613	0.995	83	APZTK62/AP33ACW/AP7DXGR
	17	*Isospora belli*	y=-3.86287x+39.08731	0.990	81	APNKRWX
	18	*Naegleria fowleri*	y=-4.11842x+41.40793	0.992	75	AP2XERK
	19	*Toxoplasma gondii*	y=-3.75486x+38.55709	0.998	85	APDJY3F
Helminths	20	*Dracunculus medinensis*	y=-3.65619x+39.48849	0.998	88	APPRVCD
	21	*Echinococcus*	y=-3.6945x+39.8014	0.997	86	APGZMAC

	22	*Schistosoma* spp.	y=-3.69378x+38.20626	0.994	87	APNKXJZ/APU69VP /APWC3FM/APXGWZJ
Quality control	23	16S rRNA gene	y=-3.61669x+37.2655	0.995	89	Ba04930791_s1
	24	18S rRNA gene	y=-3.54749x+37.74619	0.993	91	Pa99999901_s1

*The PCR primers and probes were designed by a Thermo Fisher proprietary process. And primer and probe information are stored in the Odyssey data by Thermo Fisher.

Table S4 Primers of SSU rRNA gene of *Cryptosporidium*

Primer Sequence (5'→3')	Amplicon Size (bp)
18S Primary F：TTCTAGAGCTAATACATGCG<br>18S Primary R：CCCATTTCCTTCGAAACAGGA	1325
18S Nested F：GGAAGGGTTGTATTTATTAGATAAAG<br>18S Nested R：CTCATAAGGTGCTGAAGGAGT	840

Table S5 PCR amplification systems of SSU rRNA gene of *Cryptosporidium* (50 μl)

**Reagent**	**Primary PCR (μL)**	**Secondary PCR (μL)**
ddH_2O	18.50	16.00
BSA (10 mg/ml)	1.00	-
Forward primer (10 μM)	1.25	2.50
Reverse primer (10 μM)	1.25	2.50
DNA	2.00	-
Primary PCR product	-	4.00
PrimeSTAR Max DNA Polymerase	25.00	25.00
Total volume	50.00	50.00

Table S6 Nested PCR reaction condition of SSU rRNA gene of *Cryptosporidium*

Cycle	Temperature	Duration
	94℃	5min
	94℃	30s
35x	55℃/60℃	30s
	72℃	1min/45s
	72℃	7min
	4℃	∞

Table S7 Primers of *gp60* gene *C. parvum*

Primer Sequence (5'→3')	Amplicon Size (bp)
GP60 Primary F：ATAGTCTCCGCTGTATTC	
GP60 Primary R：GAGATATATCTTGGTGCG	
GP60 Nested F：TCCGCTGTATTCTCAGCC	400
GP60 Nested R：CGAACCACATTACAAATGAAGT	

Table S8 PCR amplification systems of *gp60* gene of *C. parvum* (25 μl)

**Reagent**	**Primary PCR (μL)**	**Secondary PCR (μL)**
ddH_2O	9.4	8.9
BSA (10 mg/ml)	0.5	-
Forward primer (10 μM)	0.8	0.8
Reverse primer (10 μM)	0.8	0.8
DNA	1.0	-
Primary PCR product	-	2.0
PrimeSTAR Max DNA Polymerase	12.5	12.5
Total volume	25.0	25.0

Table S9 Nested PCR reaction condition of *gp60* gene of *C. parvum*

Cycle	Temperature	Duration
	94℃	4min
	94℃	45s
35x	59℃/59℃	20s
	72℃	15s/10s
	72℃	7
	4℃	∞

Table S10 Primers of ITS gene of *Enterocytozoon bieneusi*

Primer Sequence (5'→3')	Amplicon Size (bp)
ITS-1：GATGGTCATAGGGATGAAGAGCTT<br>ITS-2：TATGCTTAAGTCCAGGGAG	410
ITS-3：AGGGATGAAGAGCTTCGGCTCTG<br>ITS-4：AGTGATCCTGTATTAGGGATATT	392

Table S11 PCR amplification systems of ITS gene of *Enterocytozoon bieneusi* (25 μl)

**Reagent**	**Primary PCR (μL)**	**Secondary PCR (μL)**
ddH_2O	9.4	8.9
BSA (10 mg/ml)	0.5	-
Forward primer (10 μM)	0.8	0.8
Reverse primer (10 μM)	0.8	0.8
DNA	1.0	-
Primary PCR product	-	2.0
PrimeSTAR Max DNA Polymerase	12.5	12.5
Total volume	25.0	25.0

Table S12 Nested PCR reaction condition of ITS gene of *Enterocytozoon bieneusi*

Cycle	Temperature	Duration
	94℃	5min
	94℃	45s
35/30	60℃/58℃	20s/15s
	72℃	25s/20s
	72℃	10min
	4℃	∞

Table S13 High-throughput quantitative PCR results of protozoa and helminths in various processes of a drinking water plant in Shanghai

Type	ID	Target	W1	Mean (gc/L)	Range (gc/L)	W2	Mean (gc/L)	Range (gc/L)	S1	Mean (gc/g)	Range (gc/g)
protozoa	1	*Acanthamoeba genus*	-	nd	nd	-	nd	nd	33.3	1.2×10^4	nd-3.5×10^4
	2	*Acanthamoeba culbertsoni*	-	nd	nd	33.3	<LOQ	<LOQ	-	nd	nd
	3	*Acanthamoeba castellanii*	-	nd	nd	-	nd	nd	-	nd	nd
	4	*Acanthamoeba polyphaga*	33.3	4.0×10^2	nd-1.2×10^3	-	nd	nd	-	nd	nd
	5	*Balantidium coli*	-	nd	nd	-	nd	nd	-	nd	nd
	6	*Blastocystis hominis*	-	nd	nd	-	nd	nd	-	nd	nd
	7	*Cryptosporidium* spp.	-	nd	nd	-	nd	nd	33.3	<LOQ	<LOQ
	8	*Cryptosporidium parvum*	-	nd	nd	-	nd	nd	-	nd	nd
	9	*Cryptosporidium hominis*	-	nd	nd	-	nd	nd	-	nd	nd
	10	*Cryptosporidium meleagridis*	-	nd	nd	-	nd	nd	-	nd	nd
	11	*Cyclospora cayetanensis*	-	nd	nd	-	nd	nd	-	nd	nd
	12	*Enterocytozoon bieneusi*	66.7	1.9×10^4	nd-4.5×10^4	33.3	8.5×10^3	nd-2.6×10^4	33.3	<LOQ	<LOQ
	13	*Entamoeba histolytica*	-	nd	nd	-	nd	nd	33.3	1.0×10^4	nd-3.1×10^4
	14	*Encephalitozoon*	33.3	2.4×10^4	nd-7.2×10^4	-	nd	nd	-	nd	nd
	15	*Giardia* spp.	-	nd	nd	-	nd	nd	33.3	1.4×10^4	nd-4.2×10^4
	16	*Giardia lamblia*	-	nd	nd	-	nd	nd	-	nd	nd
	17	*Naegleria fowleri*	-	nd	nd	-	nd	nd	-	nd	nd
	18	*Isospora belli*	-	nd	nd	-	nd	nd	-	nd	nd
	19	*Toxoplasma gondii*	-	nd	nd	-	nd	nd	-	nd	nd
helminths	20	*Echinococcus*	-	nd	nd	-	nd	nd	-	nd	nd
	21	*Dracunculus medinensis*	-	nd	nd	-	nd	nd	-	nd	nd
	22	*Schistosoma* spp.	-	nd	nd	-	nd	nd	-	nd	nd

Table S13 High-throughput quantitative PCR results of protozoa and helminths in various processes of a drinking water plant in Shanghai

Type	ID	Target	S2	Mean (gc/g)	Range (gc/g)	S3	Mean (gc/g)	Range (gc/g)	W3	Mean (gc/L)	Range (gc/L)
protozoa	1	*Acanthamoeba genus*	66.6	5.5×10^4	nd-1.7×10^5	-	nd	nd	-	nd	nd
	2	*Acanthamoeba culbertsoni*	33.3	6.5×10^3	nd-2.0×10^4	-	nd	nd	-	nd	nd
	3	*Acanthamoeba castellanii*	-	nd	nd	-	nd	nd	-	nd	nd
	4	*Acanthamoeba polyphaga*	33.3	5.2×10^3	nd-1.6×10^4	-	nd	nd	-	nd	nd
	5	*Balantidium coli*	-	nd	nd	-	nd	nd	-	nd	nd
	6	*Blastocystis hominis*	-	nd	nd	-	nd	nd	-	nd	nd
	7	*Cryptosporidium* spp.	66.6	1.6×10^4	nd-2.7×10^4	33.3	3.8×10^3	nd-1.1×10^4	-	nd	nd
	8	*Cryptosporidium parvum*	33.3	5.6×10^3	nd-1.7×10^4	-	nd	nd	-	nd	nd
	9	*Cryptosporidium hominis*	-	nd	nd	-	nd	nd	-	nd	nd
	10	*Cryptosporidium meleagridis*	33.3	2.2×10^4	nd-6.5×10^4	-	nd	nd	-	nd	nd
	11	*Cyclospora cayetanensis*	33.3	1.5×10^4	nd-4.5×10^4	-	nd	nd	-	nd	nd
	12	*Enterocytozoon bieneusi*	-	nd	nd	-	nd	nd	-	nd	nd
	13	*Entamoeba histolytica*	-	nd	nd	-	nd	nd	-	nd	nd
	14	*Encephalitozoon*	33.3	1.9×10^4	nd-5.8×10^4	-	nd	nd	-	nd	nd
	15	*Giardia* spp.	-	nd	nd	-	nd	nd	-	nd	nd
	16	*Giardia lamblia*	-	nd	nd	-	nd	nd	-	nd	nd
	17	*Naegleria fowleri*	-	nd	nd	-	nd	nd	-	nd	nd
	18	*Isospora belli*	-	nd	nd	-	nd	nd	-	nd	nd
	19	*Toxoplasma gondii*	-	nd	nd	-	nd	nd	-	nd	nd
helminths	20	*Echinococcus*	-	nd	nd	-	nd	nd	-	nd	nd
	21	*Dracunculus medinensis*	-	nd	nd	-	nd	nd	-	nd	nd
	22	*Schistosoma* spp.	-	nd	nd	-	nd	nd	-	nd	nd

W1, reservoir water; W2, influent; W3, effluent; S1, sediment of sedimentation tank; S2, backwash water of sand filter; S3, backwash water of activated carbon filter. <LOQ indicates below detection limit under 10 L water. gc, gene copies.

Ref:

1. Ho BSW, Tam TY, Hutton P, Yam WC: **Detection and enumeration of giardia cysts in river waters of Hong Kong by flocculation-percoll/sucrose gradient-immunofluorescence method**. *Water Science and Technology* 1995, **31**(5-6):431-434.
2. Vesey G, Slade JS, Byrne M, Shepherd K, Fricker CR: **A new method for the concentration of Cryptosporidium oocysts from water**. *Journal of Applied Bacteriology* 1993, **75**(1):82-86.
3. Hashimoto A, Kunikane S, Hirata T: **Prevalence of Cryptosporidium oocysts and Giardia cysts in the drinking water supply in Japan**. *Water Research* 2002, **36**(3):519-526.
4. Nieminski EC, Schaefer FW, Ongerth JE: **Comparison of two methods for detection of Giardia cysts and Cryptosporidium oocysts in water**. *Applied and Environmental Microbiology* 1995, **61**(5):1714-1719.
5. US Environmental Protection Agency (USEPA): **Method 1623: Cryptosporidium and Giardia in water by filtration/IMS/FA**. In. Washington DC, USA: Office of Water. US Government Printing Office; 2005.
